# PsyCuraDat: Designing a User-Oriented Curation Standard for Behavioral Psychological Research Data

**DOI:** 10.3389/fpsyg.2020.579397

**Published:** 2021-01-12

**Authors:** Katarina Blask, Lea Gerhards, Maria Jalynskij

**Affiliations:** ^1^Archiving and Publication Services, Leibniz Institute for Psychology, Trier, Germany; ^2^Department of Psychology, University of Koblenz-Landau, Landau, Germany

**Keywords:** behavioral psychological research data, data sharing, curation standard, user orientation, reusability

## Abstract

Starting from the observation that data sharing in general and sharing of reusable behavioral data in particular is still scarce in psychology, we set out to develop a curation standard for behavioral psychological research data rendering data reuse more effective and efficient. Specifically, we propose a standard that is oriented toward the requirements of the psychological research process, thus considering the needs of researchers in their role as data providers and data users. To this end, we suggest that researchers should describe their data on three documentation levels reflecting researchers’ central decisions during the research process. In particular, these levels describe researchers’ decisions on the concrete research design that is most suitable to address the corresponding research question, its operationalization as well as a precise description of the subsequent data collection and analysis process. Accordingly, the first documentation level represents, for instance, researchers’ decision on the concrete hypotheses, inclusion/exclusion criteria and the number of measurement points as well as a conceptual presentation of all substantial variables included in the design. On the second level these substantial variables are presented within an extended codebook allowing for the linkage between the conceptual research design and the actually operationalized variables as presented within the data. Finally, the third level includes all materials, data preparation and analyses scripts as well as a detailed procedure graphic that allows the data user to link the information from all three documentation levels at a single glance. After a comprehensive presentation of the standard, we will offer some arguments for its integration into the psychological research process.

## Open Data and Data Sharing in Psychology

The idea that research data should be a public good, which is freely accessible to all, was first articulated by Robert King Merton in 1942 (Chignard, 2013). However, it took more than half a century for politicians and scientific organizations to recognize the importance of sharing scientific knowledge. Three important events that characterized the entry of open source and open data into Western cultures were the foundation of the Budapest Open Access Initiative, 2001, the Berlin Declaration (2003), and the Organisation for Economic Co-operation and Development (OECD) (2004). The guiding principles in these early stages of the open source and open data culture were transparency, collaboration, and participation. And even though all of these principles are at the core of good scientific practice (Gerhold et al., 2015; DFG, 2019), the routine of sharing data openly is only slowly finding its way into psychology (Dehnhard et al., 2013; Houtkoop et al., 2018; Martone et al., 2018). According to Houtkoop et al. (2018), the most prominent reasons for researchers to refrain from sharing their data are an insufficiently developed culture of data sharing as well as the perceived extra effort needed to make data reusable (see also Wallis et al., 2013).

### The Importance of Procedural Knowledge for Reusability

Indeed, “Open is not enough,” as Chen et al. (2019) expressively formulated it in their eponymous article. We need more than just open data to enable scientific reuse. Most important in this context is knowledge about the specific methods applied in the research process, i.e., procedural knowledge (Chignard, 2013; Chen et al., 2019). This is also confirmed by Blask et al. (2020b), who interviewed ten researchers in psychology with regard to what they think is needed for an optimized reuse from their perspective as users and providers of research data. All interviewees stated that reuse of psychological research data is critically dependent on a profound understanding of the underlying procedure. However, they differed in their assumptions on how this might be achieved. While almost all of them demanded an extensive and comprehensible codebook, they diverged with regard to the provision of additional procedural information that may be, for instance, achieved via a study protocol or data preparation and analysis scripts.

This disagreement among researchers on best practices for documenting psychological research data, especially those belonging to the long tail of science, may stem from a lack of procedural regularity (cf. Hertwig and Ortmann, 2001). Considering, for instance, experimental procedures in social, developmental, or organizational psychology, it quickly becomes clear why it is often so difficult to replicate the empirical results from those studies. In essence, many experimental procedures used in these sub-disciplines of psychology lack some critical features essential to the replicability of empirical findings. These are, among others, repeated trials and script enactment, the former allowing participants to get used to the experimental procedure, and the latter reducing participants’ uncertainty in the experimental setting (cf. Hertwig and Ortmann, 2001). Oftentimes these features are not implemented in the experimental procedure because they counter the effect to be investigated. For instance, if a study is targeted at the investigation of one-trial learning (e.g., Öhman et al., 1975), repeated trials would disqualify the study as an instance of one-trial learning. However, these features, essential to the replicability of empirical findings, can be added to psychological procedures by transferring them to the data resulting from their application. On the one hand, the probability to obtain consistent results across studies aimed at investigating the same research question, but using new data or other computational methods increases to the extent that data are reusable. On the other hand, the reusability of data can be improved by providing a detailed description of the experimental procedure (i.e., an equivalent to script enactment during the data collection process). Within this framework, the responsibility for the replication of empirical findings no longer exclusively lies in the hands of the individual researcher, but in the hands of the psychological community.

Despite this relatively straightforward approach to the reusability and the related replicability of research data, there does not seem to be a single standard at present on which psychologists can rely to make the procedural knowledge associated with their data accessible to other researchers. Instead, there exists a host of standards, discipline-specific and -unspecific, that allow for the formal specification of a dataset. One prominent discipline-specific standard is, for instance, the BIDS (the Brain Imaging Data Structure; Gorgolewski et al., 2016) – a standard for describing and organizing magnetic resonance image (MRI) datasets (see also Niso et al., 2018 and Pernet et al., 2019 for a BIDS specification of MEG and EEG data, respectively). Moreover, psychologists might use the metadata standard from the Data Documentation Initiative DDI (DDI 3.2) that was developed to describe social, behavioral and economic data (cf. Dehnhard et al., 2013). However, this standard is so far away from common practices in psychological research that it is very difficult for researchers to integrate it into their daily routines. Discipline-unspecific standards – mainly suited for the bibliographic documentation of datasets – are, among others, the Dublin Core and the Darwin Core metadata standard (Weibel et al., 1998; Wieczorek et al., 2012). However, the majority of psychological researchers use neither discipline-specific nor -unspecific standards because they are largely unaware of them. In a recently conducted online survey on the reuse and reusability of psychological research data (Blask et al., 2020a), it turned out that only four out of 57 psychologists (i.e., 7%) were familiar with the BIDS and the DDI standard, three knew about Dublin Core (i.e., 5.2%) and no one was aware of Darwin Core. Similarly, the results of a survey conducted by the Stanford Center for Reproducible Neuroscience – Medium (Feingold, 2019) showed that only 15% of the 116 surveyed researchers from the neuroimaging community used the BIDS to document their data.

### The Pros and Cons of Using Curation Standards

But why are these standards not adopted by psychologists, especially those that have been developed with the help of psychologists, like the BIDS? The answer to this question can be approached from two perspectives, the first being the psychologist as a data user and the second being the psychologist as a data provider. Considering the low uptake of metadata standards in psychology from the perspective of a data user, one could hypothesize that researchers who reuse data are still in the minority. Therefore, the quest for well-documented data and thus the need for a common curation standard might be rather low. A look at the percentage of re-analyses undertaken in psychological research in the recent past confirms this: We conducted a short research on the development of the proportion of re-analyses out of all empirical studies published between 1980 and 2018 in order to get a more precise overview of actual reuse in psychology^1^. When considering the proportion of re-analyses over 10-year intervals from 2000 to 2018 (before 2000 there were almost no re-analyses), it turns out that empirical studies involving re-analyses of existing data almost tripled. Even though the overall proportion of re-analyses is still very low with only 0.48% in the period from 2010 to 2018, the amount of re-analyses seems to increase continuously. Therefore, improving the reusability of psychological research data is essential for further accelerating the scientific progress in psychology (cf. Hardwicke et al., 2018). This is not least important if you take into account that probably the most common reason for reusing data is not re-analysis but meta-analysis, where researchers might have to rely on many datasets.

Taking the perspective of a data provider, there are multi-faceted reasons for not sharing data ranging from legal concerns (e.g., the violation of participants’ privacy rights), a personal sense of ownership, the fear of “scooping” (Longo and Drazen, 2016) and reputation loss (Martone et al., 2018), all the way to the more infrastructural reasons like a lack of incentives (Wallis et al., 2013; McKiernan et al., 2016) or discipline-specific research data centers. One of the most prominent reasons for not using data curation standards is the generally perceived extra effort associated with data management (Blask and Förster, 2018; Pronk, 2019). This perception may be held by researchers because they are unfamiliar with the corresponding standards or because they do not feel well-equipped to use them (Tenopir et al., 2015). Hence, two reasons for psychological researchers’ failure to use data curation standards (e.g., metadata standards) are likely an insufficient knowledge of these standards as well as the existing standards’ lack of usability. Of course, a number of very good handbooks and tools on reproducible and transparent data science are available, for instance the Turing way (The Turing Way Community et al., 2019) or GitHub. Likewise, there are information platforms^2^^,3^ as well as tools and services (e.g., Strasser et al., 2014) that might help to make these standards an integral part of psychologists’ general knowledge of scientific methodology. However, the core issue is that a curation standard for describing research data should be easily applicable and integrable. Even then, the application of these standards remains a time-consuming and sometimes also tedious process. As we suggest, this is exactly where the crux lies. Integrating the data curation process into the research process ameliorates both issues: A thorough documentation of all decisions made during the research process, which is also proposed in the preregistration literature (e.g., Nosek et al., 2019), not only furthers the reusability and reproducibility of scientific data. It also relieves researchers of the burden of a time-consuming reconstruction of the research process at the end of their project (cf. Weichselgartner, 2017). A further advantage of data curation is that similar to preregistrations, providing a thorough documentation of one’s research data can positively affect one’s career (McKiernan et al., 2016). In summary, we argue that the benefits of applying a data documentation standard should always outweigh the potential costs. Given that the reusability of scientific data cannot be reached by merely sharing them (Chen et al., 2019) but that a sophisticated description is needed to understand and reuse them, applying a standard like ours for documenting, curating and managing research data is definitely worth the effort. In order to make researchers use a standard and profit from the latter’s application, the standard has to be suitable for documenting researchers’ decisions during the research process in a comprehensive and efficient way.

## PsyCuraDat: Creating a User-Oriented Data Curation Standard

The BMBF-funded project PsyCuraDat ties into this highly topical conversation. The main goal of the project is to develop a user-oriented data curation standard that helps psychological researchers to either provide or understand the procedural knowledge associated with a given dataset. To achieve this goal, PsyCuraDat has pursued a similar methodological approach as the BIDS (Gorgolewski et al., 2016). That is, similar to the BIDS, PsyCuraDat aims at developing its standard with the help of the community. Specifically, researchers were asked in an online survey which procedural information about a dataset they would need to reuse it for different purposes (e.g., meta-analyses, re-analyses, systematic review, illustrations, etc., for a more detailed description of the procedure see Blask et al., 2020b). The entirety of the queried procedural knowledge was derived from the Journal Article Reporting Standards (JARS) published by the APA (APA Publications and Communications Board Working Group on Journal Article Reporting Standards, 2008). The results of the survey indicate that the majority of researchers would welcome a curation standard requiring the enrichment of psychological research data with information on the research design, the concrete operationalization as well as on the conducted primary and additional analyses. Thus, in order to foster reusable research data, curation standards should provide clear guidelines on how to enrich research data with information on the data collection and data analysis process. While more information on the data analysis process can be easily provided in the form of an analysis script – including a documentation of all analysis steps –, a comprehensive description of the data collection process can be a big challenge. This challenge arises from the necessity to describe data in the context of the underlying study plan or research design, respectively. That is, we have to tell the whole story of the research data according to the research process (cf. Surkis and Read, 2015).

In this article, we want to propose a curation standard for behavioral psychological research data that allows researchers to tell the whole story about their data by relying on the specific methodological requirements of the psychological research process and the decisions inherent in this process. Methods, in this context, are generally defined as procedures that are used to gather scientific evidence (Bierhoff and Petermann, 2014). The defining features of psychological research methods are essentially a detailed process description of the research process and the research design. While a detailed process description helps to put empirical evidence into practice, the research design describes the methodological approach that underlies the research process (cf. Bierhoff and Petermann, 2014). This methodological approach in turn can be described on a construct level and on an operational level, the latter concerning the measurement of the theoretical constructs (Mayntz et al., 1969). Translating these core features of psychological methods into decisions that researchers have to make, one could argue that a method-oriented curation standard should allow for a comprehensive description of the following three decisions: researchers’ decisions related to the conceptual considerations associated with a specific research design, the operationalization of the research design, and all additional steps taken during the data collection and analysis process.

Based on these considerations, we propose the curation of psychological research data on three different levels. The first level is representative of the research design on a construct level and includes information on the number of measurements, inclusion/exclusion criteria, population, sampling method, sample size, power, assignment methods (e.g., random or non-random), control operations, setting, methodological approach, variables included in the design (i.e., independent, dependent, control, and external variables), and a precise description of the hypotheses. In sum, this level is intended to provide a comprehensive understanding of the data at the study level.

On the second level, the research design is described on an operational level. To this end, researchers describe which methods they used to operationalize the variables that have been described on the first level. That is, for all events constituting the applied procedures, i.e., for all substantial data – variables described on the first level –, the following aspects are provided: name, label, name of the procedure, values, scale level, and information about the coding of missing values. This level allows to understand researchers’ decisions on how to translate the conceptual research design and the associated hypotheses into data, which can be used to test these hypotheses.

**FIGURE 1:**
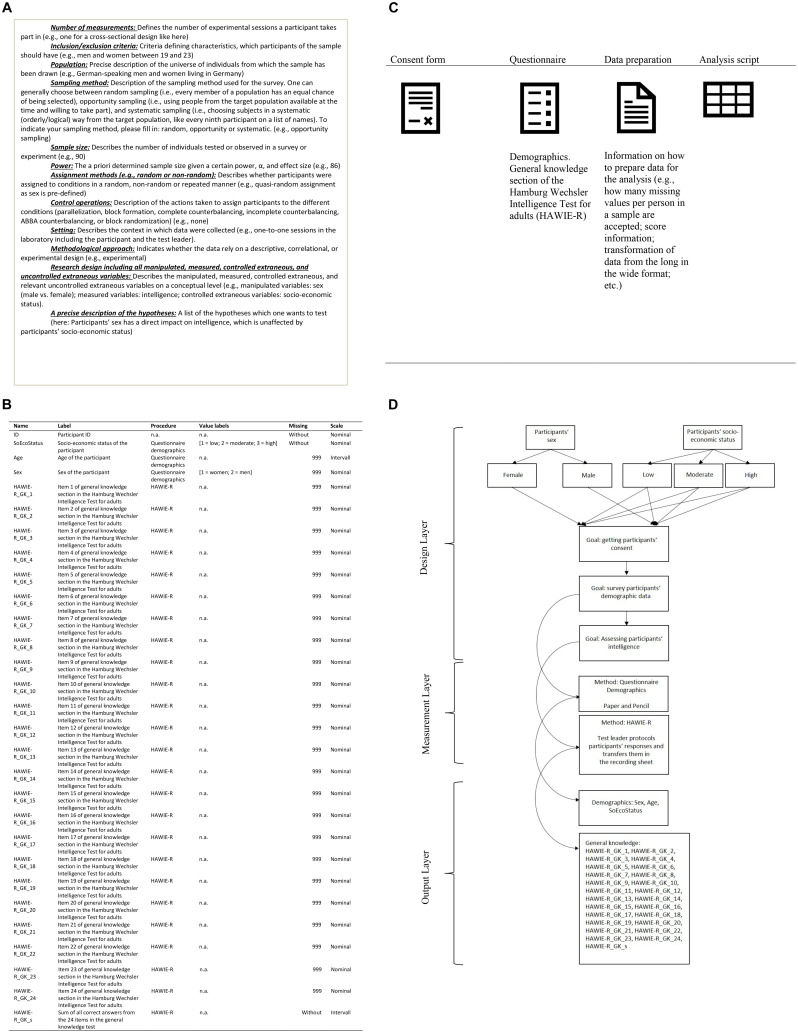
This is an exemplary depiction of a typical documentation following the PsyCuraDat standard. The first part **(A)** includes a short definition of all elements that have to be included in the read-me file on the first documentation level as well as a corresponding example, presented in brackets after each definition. The second part **(B)** includes an exemplar of a codebook as it might be structured in this exemplary study. The third part **(C)** refers to the third documentation level and hints at the necessity of providing all relevant materials. Finally, the fourth part of the figure **(D)** depicts a procedure graphic representing the whole data collection process and allowing for the linkage of information from all three documentation levels. **(A)** Creation of a read-me file including the conceptual description of the research design. **(B)** Creation of a codebook describing all substantial variables. **(C)** Providing all relevant materials (in the present example, these might be the consent form, the survey protocol, the questionnaire containing the demographic items, the data preparation and analysis scripts). **(D)** Providing a procedure graphic.

On the third level, researchers provide a detailed description of the whole data lifecycle, including the data collection and analysis process. This level comprises all materials relevant to a profound understanding of these processes (e.g., program code, consent form, stimulus material, survey protocol, conceptual description of all data preparation, and analyses scripts) and helps researchers to understand data not only on a superficial study or dataset level, but in the context of their lifecycle. To link information from the first two levels to those from the third level in a comprehensive way, level three also contains a graphic representation of the procedure. The procedure graphic, optimally realized within a flow-chart, comprises on a design layer a short description of all phases from the data collection process, a phase being defined by a distinct survey goal. The formal presentation of the different phases should allow for a clear mapping of the conditions described in the research design on the first documentation level and the different procedural phases. If all phases have been described with regard to their specific goal, a clear description of the survey method and its’ associated medium (e.g., the used EEG machine including EEG headset and relevant software/hardware, the version of the experimental software, paper and pencil, etc.) follows on a measurement layer. The latter, though, is only applied to phases targeted at the operationalization of the substantial variables included in the dataset. To allow for a facilitated referencing from information provided for the different methods and those provided earlier in the documentation process, labeling of the described methods should reflect the name of the procedure provided on the second level. Finally, participants’ reactions within the different phases are displayed in an output-layer. In particular, the names of the corresponding substantial data as described on the second level are listed and separated according to their purpose. For an exemplary presentation of such a procedure graphic for a 2 × 3 between-subjects design, see Figure 1.

The formal provision of information from the first level takes place via a read-me file and information from the second level is provided via an extensive codebook (UTF 8-coded text file, e.g., ^∗^.csv). The graphic description of the procedure on the third level can be provided in the common formats for high-resolution pictures, i.e., ^∗^.tif, ^∗^.png, ^∗^.jpg. All other materials should be – wherever possible – provided in non-proprietary formats. The conceptual description of all data preparation and analysis steps should be provided as another read-me file. An exemplary presentation of the whole documentation process is displayed in Figure 1.

## Discussion: Integrating the User-Oriented Curation Standard Into the Research Process

Of course, describing a dataset on all three levels may be very time-consuming, especially with regard to the second level. An attentive reader might say this standard may solve the problem of researchers who want to reuse data, but not of researchers providing data. However, this is only true at first glance. Indeed, researchers already document most of this information, but not in a systematic way that would allow other researchers a more comprehensive view on their data. For instance, many researchers define their population as well as inclusion and exclusion criteria when making use of a subject-pool system. Likewise, all other information from the first level as well as the extensive codebook and information included in the graphic overview, which are provided on the second and third level are defined during the preparation of the data collection process (e.g., for programming or also a preregistration of the study). Of course, this information might have to be updated during the research process if plans change, for instance due to new findings or methods. Thus, documenting data in accordance with the proposed curation standard, is mainly about rearranging the recording of one’s own research process and can be done in parallel with the conventional tasks within this process. Furthermore, it could contribute to a better comprehensibility and reusability of a researcher’s own data. For example, if a researcher needs to handle several projects at a time – which should be the case for the majority of researchers – or wants to take a glance at a research project retroactively after some years, a previous documentation may be helpful.

Likewise, it should be fairly simple for researchers to describe their data according to the third level when all steps of the research process are done. Note that we are not suggesting that for instance creating a graphic overview is not time-consuming and thus costly. However, the graphic overview of the procedure can already be prepared during the data collection process and can then later be used within the associated publication. In a similar vein, a conceptual description of all data preparation and analysis steps can already be prepared with the help of test data generated during the preparation of the data collection process. Finally, and in order to make the whole research process comprehensible and transparent, researchers should provide at least a description of all the materials that they used during the data collection process. In this case, the comprehensibility of the data is preserved and if researchers want to replicate these findings they can contact the authors.

Thus, in order to support research practices in psychology that are defined by a high degree of research integrity and, in particular, transparency, we propose that data should be curated on all three levels. Ideally, the documentation process is complemented with the preparation and submission of a preregistration, which is another means to further transparency during the research process. In this way, the proposed theoretically developed curation standard can perhaps help to overcome some of the “methodological shortcomings” of psychological research (e.g., p-hacking, HARKing, or even conscious fraud). Moreover, the application of this standard throughout the whole research process might also reduce the costs for archiving, maintaining and updating this information in a disciplinary data repository. Indeed, the submission to a disciplinary data repository should be facilitated by the comprehensive documentation of one’s data because metadata and codebook, for instance, are already existing in the expected form. Similarly, the maintenance and updating of information should be facilitated if all parties involved in the data curation process (i.e., researchers and infrastructure providers) adhere to the same standard. At the beginning, this could be complex for some researchers because of novelty or changes in their own documentation strategies during the research process. However, after a while, a common standard should accelerate the whole data curation process and allow researchers to use the full potential of psychological research data.

## Conclusion

Against the background of the still ongoing debate on insufficient data sharing in psychology, the present article introduces a user-oriented curation standard for behavioral research data in psychology. The proposed standard is taking into account the quest of psychological researchers for the procedural knowledge associated with a given dataset by providing information on the key characteristics of psychological methods. Namely, it allows for the description of the research design and its operationalization as well as a detailed description of the associated data collection and analysis process. The usability of the current curation standard arises from its strong orientation toward the peculiarities of the psychological research process. Its usefulness is based in its potential to overcome some methodological shortcomings in psychology that are causal for the often discussed replicability crisis in psychology (cf. Hertwig and Ortmann, 2001). In particular, the proposed standard can help to increase the reusability of psychological research data and thereby allows for the conduct of a community-driven test of their replicability.

Of course, some researchers might argue that the application of such a standard is not feasible because it is too time-consuming. However, if researchers want to tell the community the whole story of their data and want their findings to be replicated or reproduced by other researchers, they should integrate this standard into their common research process. To optimize the integration of the standard into the research process, future research should empirically verify the positive impact on the reusability of data curated in accordance with the proposed standard. Relevant studies are planned in the frame of the PsyCuraDat project but should also be conducted by independent research groups.

## Author Contributions

The initial idea for the standard was conceived by KB and further refined with the help of LG and MJ. All authors contributed to writing the manuscript, and approved the final version of the manuscript for submission and take responsibility for its content.

## Conflict of Interest

The authors declare that the research was conducted in the absence of any commercial or financial relationships that could be construed as a potential conflict of interest.

## Footnotes

1 Note that we only analyzed empirical studies indexed in PSYNDEX (www.psyndex.de/) at the point of the investigation and that we cannot account for the repeated analysis of one dataset or the distributed publication of findings across multiple studies.

2 https://rdmtoolkit.jisc.ac.uk

3 https://www.fosteropenscience.eu
